# Author Correction: Hidden paths to endless forms most wonderful: ecology latently shapes evolution of multicellular development in predatory bacteria

**DOI:** 10.1038/s42003-022-04312-w

**Published:** 2022-12-07

**Authors:** Marco La Fortezza, Olaya Rendueles, Heike Keller, Gregory J. Velicer

**Affiliations:** 1 Institute for Integrative Biology, ETH Zurich, Universitätstrasse 16, 8092 Zurich, Switzerland; 2 Microbial Evolutionary Genomics, Institut Pasteur, CNRS, UMR3525, 75015 Paris, France

**Keywords:** Experimental evolution, Evolutionary developmental biology

Correction to: *Communications Biology* 10.1038/s42003-022-03912-w, published online 16 September 2022.

In the original version of the Article, the Acknowledgements incorrectly referenced “grant 31003B_6005”. The text should read “grant 310030B_182830”. This has now been corrected in the PDF and HTML versions of the Article.

