# Supplementary material for: Hidden paths to endless forms most wonderful: ecology latently shapes evolution of multicellular development in predatory bacteria
Source: Commun Biol. 2022 Sep 16;5:977. doi: 10.1038/s42003-022-03912-w (PMC9481553; doi:10.1038/s42003-022-03912-w)
Supplement: Supplementary file 2 — Supplementary Information [file 42003_2022_3912_MOESM2_ESM.pdf]

SUPPLEMENTARY INFORMATION

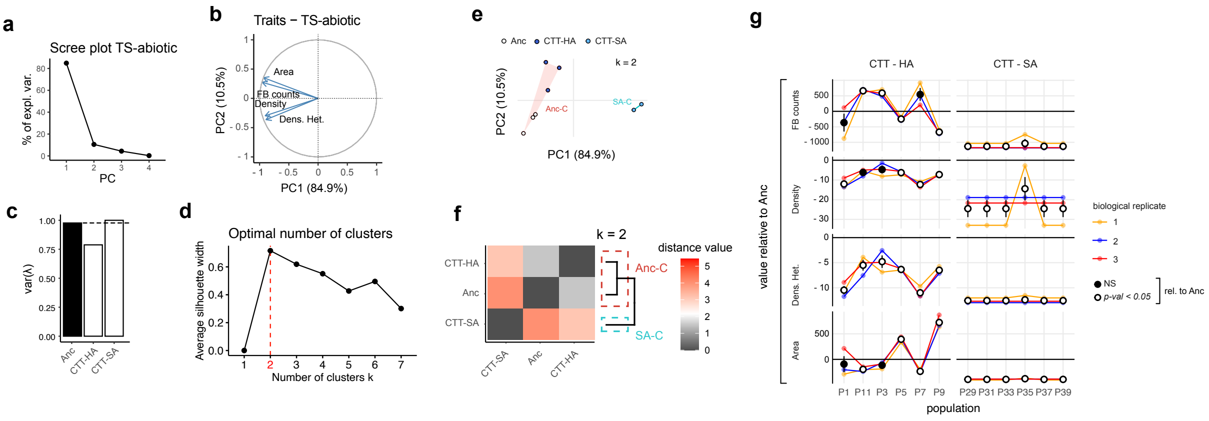

Supp. Fig. 1. PCA, k-means, phenotype-dendrogram and population-level single traits analyses of TS-abiotic.

a, Scree plot indicating the percentage of variance explained by each principal component obtained from the PCA run on TS-abiotic and Anc together. **b**, Distribution in multivariate space of eigenvectors representing the four morphological traits used in the PCA. **c**, Correlation levels of the four morphological traits estimated for each treatment relative to the ancestral level (1.0, dashed line), as reflected by eigenvector variance (λ). **d**, Optimal number of clusters (k = 2) obtained from the average silhouette width (see *Methods*: *Cluster analysis*) of PCA results. **e**, Subdivision of all individual replicates (Anc: open circles, CTT-HA: dark blue circles, CTT-SA: light blue circles) by the two clusters Anc-C (Ancestor-Cluster, red-shaded region) and SA-C (Soft Agar-Cluster, blue-shaded region) obtained from the k-means cluster analysis (k = 2, see *Methods: Cluster analysis*). **f**, Heatmap of pairwise Euclidean distances measured between individual centroids mapped on the multivariate space (Fig. 1b). Individual tails color ranging from 0 (grey) to 5 (red) indicate distance values. Dendrogram of hierarchical clustering (see *Methods: Cluster analysis*) across all pairwise Euclidean distances is reported on the right side. To facilitate the contrast between the two clustering methods, k-means clusters Anc-C (red-dashed box) and SA-C (light-blue dashed box) (see **e**) were superimposed on the dendrogram. **g**, Single developmental trait values relative to Anc (horizontal-black line set at zero) of all single evolved populations from TS-abiotic. Values were averaged across three independent biological replicates, which are each represented by small colored circles with connecting lines, (n = 3). Average values (large circles) are reported with the associated SEM in all cases. Colored lines connect different population values measured within the same biological replicate. In both panels, white and black filled circles indicate average values with a significant or non-significant difference from the Anc, respectively. In **g**, significance levels were calculated with one-way ANOVA followed by two-tailed Tukey tests. *p*-values of all comparisons of evolved treatments with Anc, as well as all pairwise comparisons between evolved treatments are reported in **Data Supp. Fig. 1**.

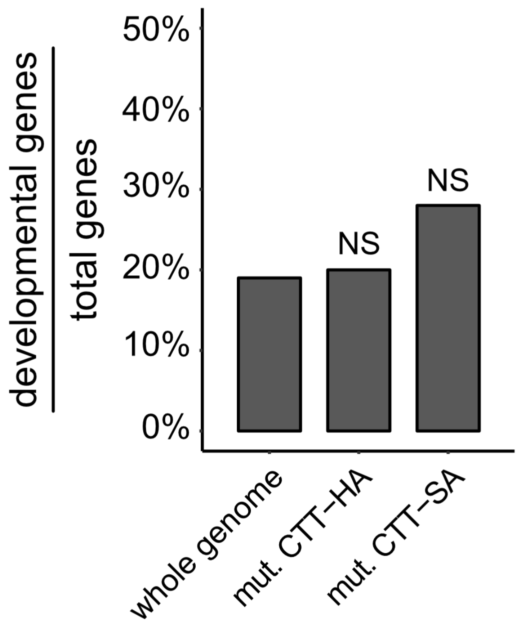

Supp. Fig. 2. Developmental genes are not over-represented among mutated genes in the CTT-HA and CTT-SA treatments.

Bar graph showing the proportion of all annotated *M. xanthus* genes designated as developmental genes [ref. 84] compared to the proportion of developmental genes among genes mutated in the MyxoEE-3 cycle 40 CTT-HA and CTT-SA evolved populations [ref. 79] (Significance was tested with Fisher's exact test for multiple comparisons; NS = not significant). **Data Supp. Fig. 2**.

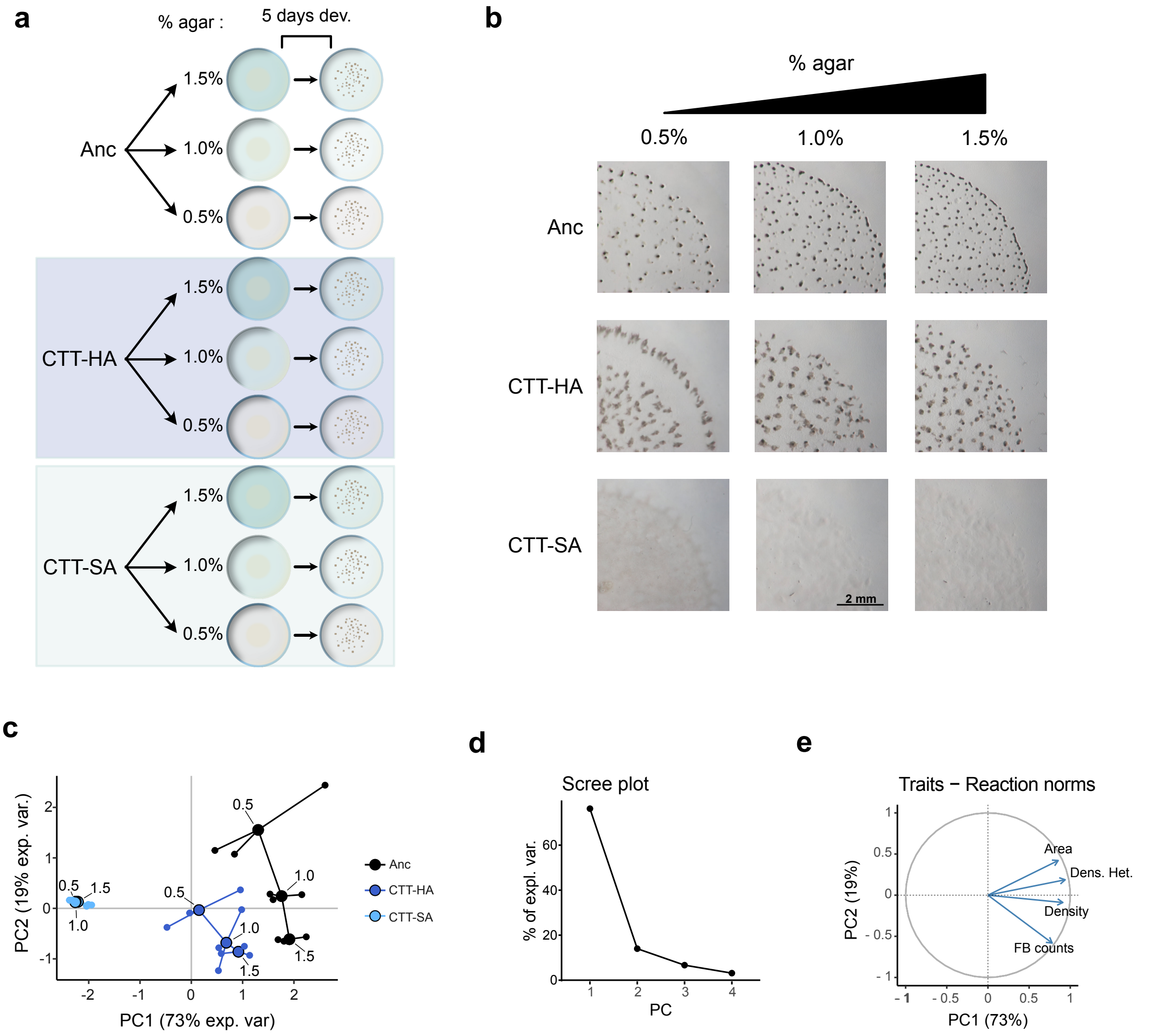

Supp. Fig. 3. Reaction-norm analysis: design, representative images and PCA.

a, Schematic representation of the experimental procedure used to analyze developmental plasticity across the Anc clones and TS-abiotic evolved populations. Following the protocol to induce fruiting body development (see *Methods: Induction of development*), cells from all evolved populations of both CTT-HA and CTT-SA selective environments and all Anc sub-clones were plated on TPM starvation plates that differed only in agar concentration along a gradient (0.5%, 1.0% and 1.5%). After five days of starvation, populations were morphologically analyzed. **b**, Images of developmental plates after five days of starvation (CTT-HA = P3; CTT-SA = P39) (scale bar = 2 mm). **c**, PCA of developmental reaction norms across all three agar concentrations. Large circles depict the centroids of three independent replicates (small circles and connecting lines). Anc, CTT-HA and CTT-SA multivariate reaction norms are shown in black, dark blue and light blue, respectively. The x and y axis labels report the percentage of variance explained by the two principal components PC1 and PC2, respectively. **d**, Scree plot indicating variance percentages explained by the four principal components. **e**, Eigenvectors of the four developmental traits used in the PCA. Data Supp. Fig. 3.

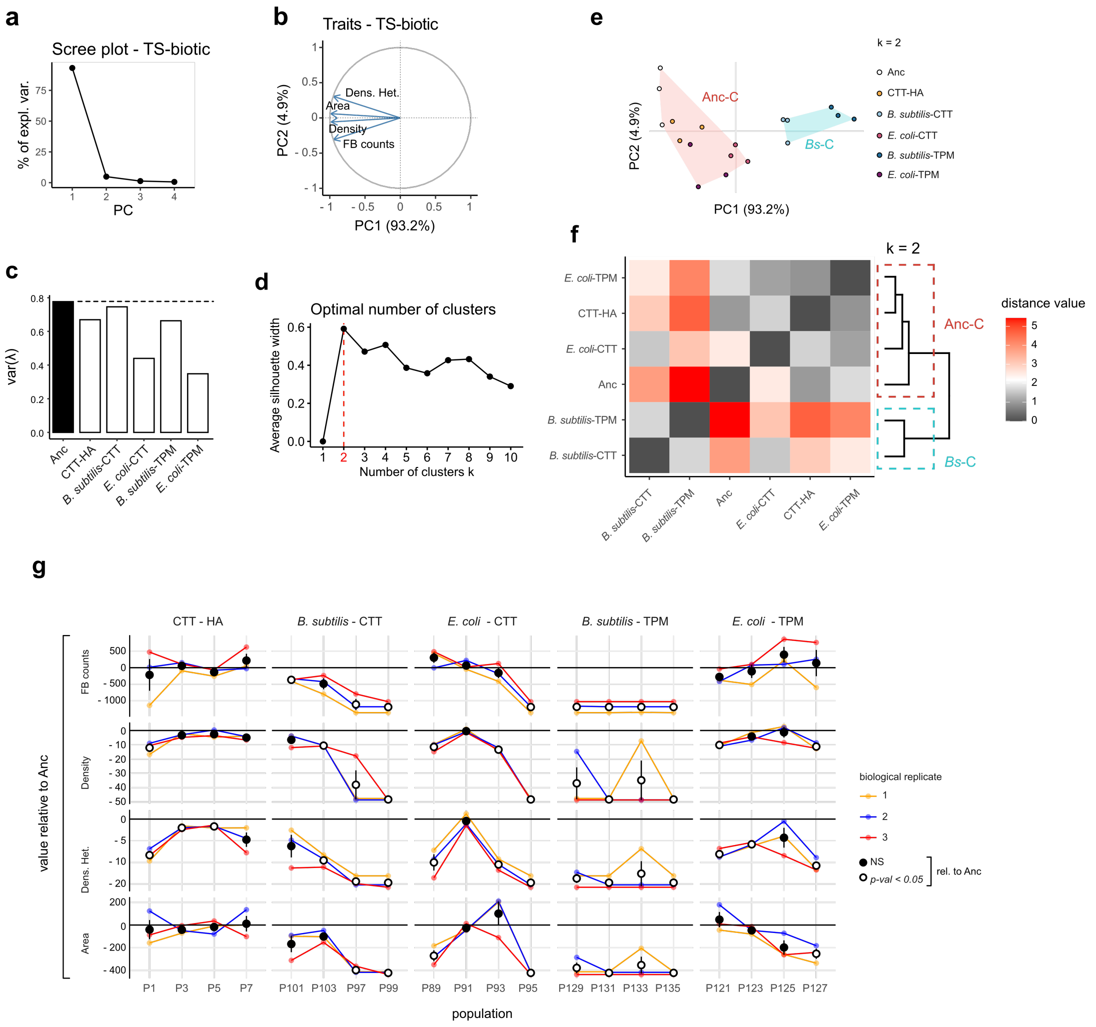

Supp. Fig. 4. PCA, k-means, phenotype-dendrogram and population-level single traits analyses of TS-biotic.

a, Scree plot indicating the percentage of variance explained by each principal component obtained from the PCA run on TS-biotic and Anc together. **b**, Eigenvectors representing each of the four morphological traits used in the TS-biotic PCA. **c**, Correlation levels of the four morphological traits estimated for each treatment relative to the ancestral level (1.0, dashed line), as reflected by eigenvector variance (λ). **d**, Optimal number of clusters (k = 2, highlighted in red on the x-axis) obtained from the average silhouette width (see *Methods: Cluster analysis*) of the TS-biotic PCA results. **e**, Subdivision of all individual replicates by the two optimal statistical clusters Anc-C (Ancestor-cluster, red-shaded region) and Bs-C (*B. subtilis*-cluster, blue-shaded region) obtained from the k-means cluster analysis (k = 2, see *Methods: Cluster Analysis*). **f**, Heatmap of pairwise Euclidean distances measured between individual centroids mapped on the multivariate space (Fig. 1b). Individual tail colours ranging from 0 (grey) to 5 (red) indicate distance values. Dendrogram of hierarchical clustering (see *Methods: Cluseter analysis*) across all pairwise Euclidean distances is reported on the right side. To facilitate the contrast between the two clustering methods, k-means clusters Anc-C (red-dashed box) and Bs-C (light-blue dashed box) (see **e**) were superimposed on the dendrogram. **g,** Values of individual developmental traits relative to Anc (horizontal-black line set at zero) of all single evolved populations from TS-biotic selective environments. Values were averaged across three independent biological replicates, which are each represented by small colored circles with connecting lines (n = 3). Average values (large circles) are reported with the associated SEM in all cases. Colored lines connect different population values measured within the same biological replicate. In both panels, white and black filled circles indicate average values with a significant or non-significant difference from the Anc, respectively. In **g**, significance was calculated in all cases with one-way ANOVA followed by two-tailed Tukey tests. *p*-values of all comparisons of evolved treatments with Anc, as well as all pairwise comparisons between evolved treatments are reported in Data Supp. Fig. 4.

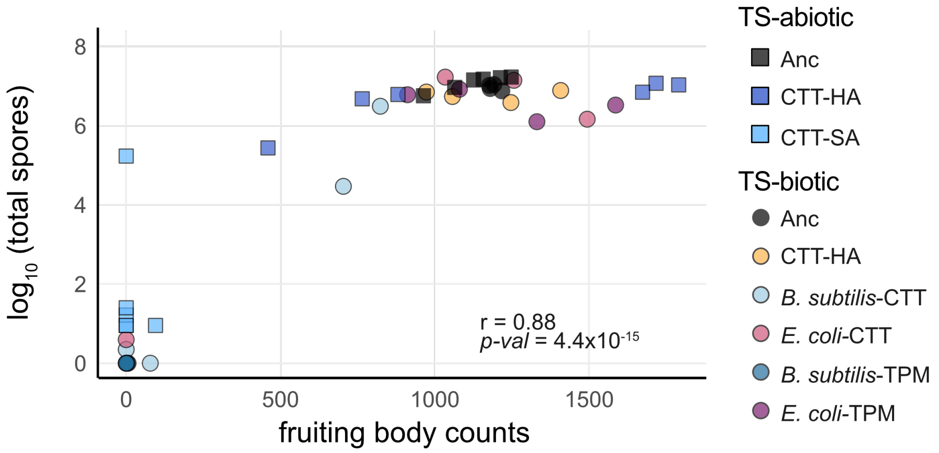

Supp. Fig. 5. Fruiting body counts and spore counts correlate positively across all TS-A and TS-B populations and their ancestors.

Scatterplot showing the relationship between average fruiting body counts and the averaged log_{10}-transformed spore counts across the three independent biological replicates for each individual TS-abiotic and TS-biotic evolved population and the Anc sub-clones (n = 42). Pearson's correlation (r) and the associated *p*-value are shown inside the plot area. **Data Supp. Fig. 5.**

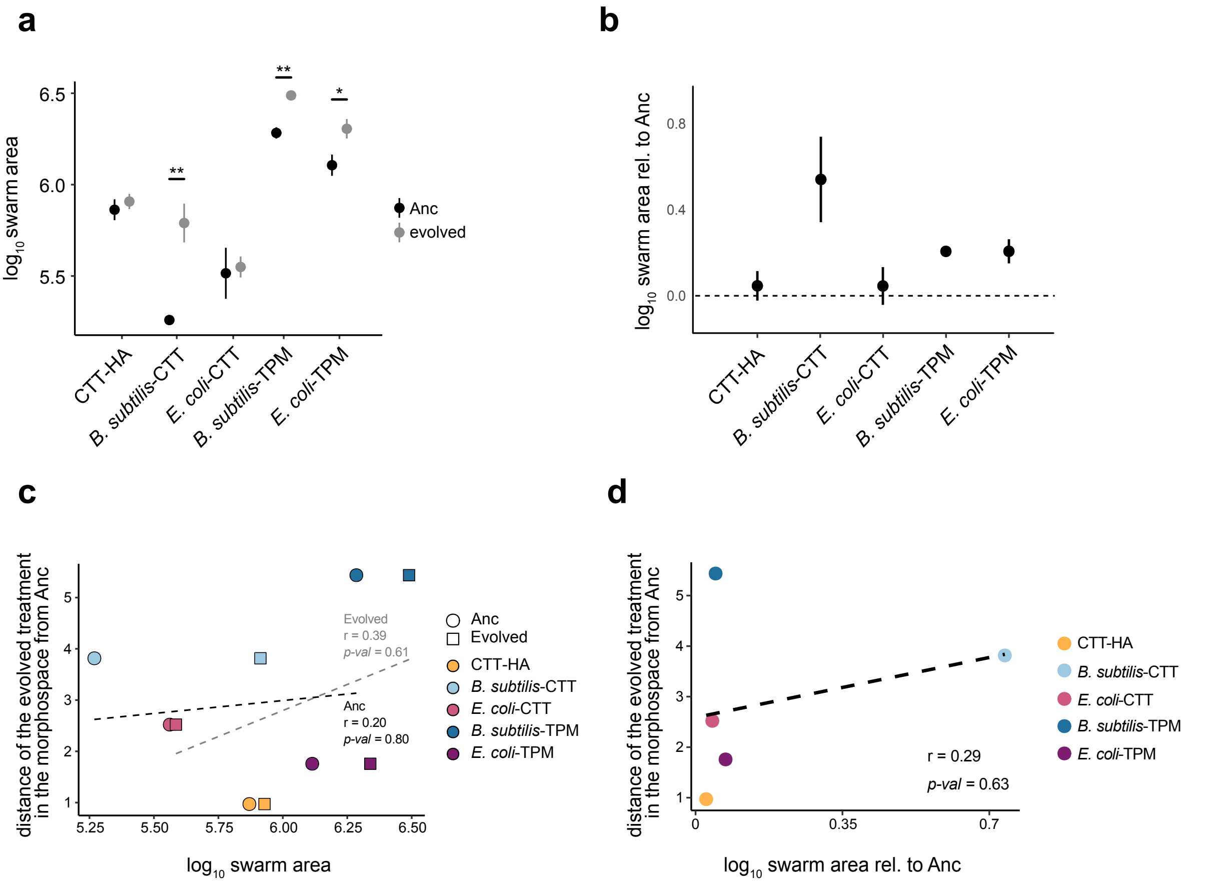

Supp. Fig. 6. The degree of treatment-level developmental LPE does not correlate with either absolute selective-environment swarming rates during MyxoEE-3 or the degree of evolutionary change in swarming rates.

a, Treatment-level average swarming areas of TS-biotic evolved populations (grey dots) assayed in their original selective environments (see Fig. 1b) compared to Anc (black dots). Asterisks indicate significant differences between Anc and evolved values (two-sided *t*-test with Welch correction). *p*-values are reported in Data Supp. Fig. 5. **b,** Log-scale degree of evolutionary change in swarming rate for each TS-biotic treatment is shown by subtraction of (log-transformed) ancestral values from evolved values (black dashed line set at 0 indicates ancestral level). For both **a** and **b,** error bars indicate SEM for variation in per-replicate treatment-level averages calculated across biological replicates (n = 4). **c**, Scatter plot of Anc (circles) and average per-treatment evolved (squares) swarm areas in the five respective TS-biotic MyxoEE-3 environments vs developmental-morphospace distance of evolved treatments from Anc. Dashed lines are regression lines calculated for the Anc (black-dashed line) and evolved populations (gray-dashed line). Pearson's correlation values (*r*) and the associated *p*-values are reported within the plot (black and gray texts refer to the data from Anc and evolved populations respectively). **d,** Scatter plot of swarm area relative to the Anc (values from panel B) vs treatment-level morphospace distance from Anc (Fig. 4b and Supp. Fig. 5f). All data and *p*-values can be found in **Data Supp Fig. 6**.

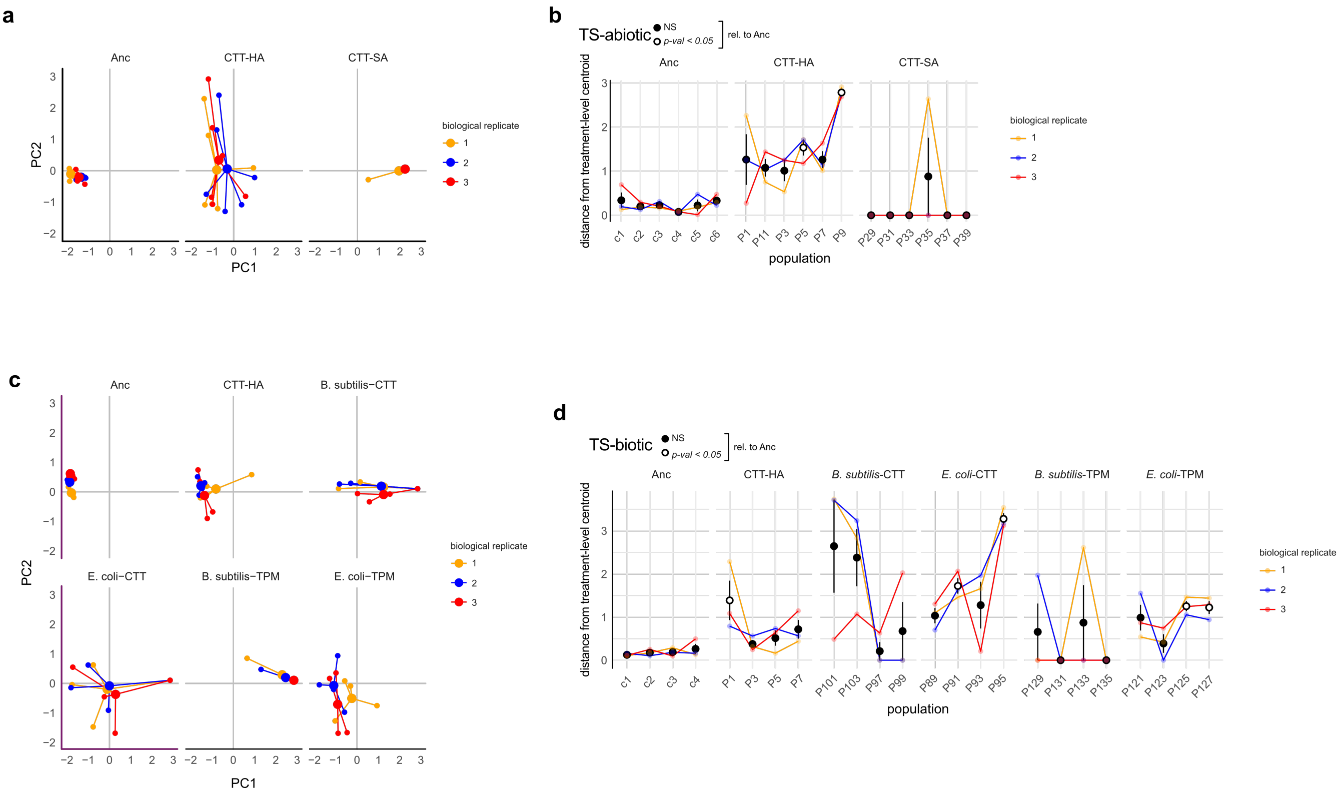

Supp. Fig. 7. PCA-dispersion analyses among individual populations

a and **c,** Morphospace showing the phenotypic distance (line lengths) of each population (small circles) from the averaged centroid (large circles) of all populations from the same treatment per replicate for TS-abiotic (**a**) and TS-biotic (**c**) treatments. The three independent replicates are identified by color (orange, blue, and red). **b** and **d**, Quantitative plots (y axis) of the phenotypic distances reflected by line lengths in panels **a** and **c**. Average values per population across replicates are reported (large circles) with the associated SEM (n = 3). Colored lines connect values of different populations measured within the same biological replicate (same color). For both panels **b** and **d**, white and black filled circles indicate average values with a significant or non-significant difference from the Anc average value, respectively (Significance was calculated in all cases with one-way ANOVA followed by two-tailed Tukey test). p-values of all comparisons of evolved treatments with Anc, as well as all pairwise comparisons between evolved treatments are reported in Data Supp. Fig. 7.

Supp. Table 1. List of the MyxoEE-3 treatments relevant to this study (adapted from Supp. Table 2 in ref. 79)

			Examined populations (P) organized by ancestral GJV1 (Anc) sub-clone					
**Treatment**	**Description**	**MyxoEE-3 cycle**	GJV 1.1[a]	GJV 1.2	GJV 1.3	GJV 1.4	GJV 1.5	GJV 1.6
Treatment Set abiotic (TS-abiotic)								
CTT hard agar (CTT-HA)	1% Casitone, 1.5% agar	40	P1	P3	P5	P7	P9	P11
CTT soft agar (CTT-SA)	1% Casitone, 0.5% agar	40	P29	P31	P33	P35	P37	P39
Treatment Set biotic (TS-biotic)								
CTT-HA	1% Casitone, 1.5% agar	18	P1	P3	P5	P7		
E. coli-CTT-HA here '*E. coli*-CTT'	*E. coli* grown on CTT HA	18	P89	P91	P93	P95		
B. subtilis-CTT-HA here '*B. subtilis*-CTT'	*B. subtilis* grown on CTT HA	18	P97	P99	P101	P103		
E. coli-TPM-HA here '*E. coli*-TPM'	*E. coli* overlaid on TPM HA	18	P121	P123	P125	P127		
B. subtilis-TPM-HA here '*B. subtilis*-TPM'	*B. subtilis* overlaid on TPM HA	18	P129	P131	P133	P135		

[a] GJV1.1-GJV1.6 are labeled as c1-c6 in Supp. Fig. 7b, and 7d.

Supp. Table 2. Experimental differences between TS-biotic treatments during MyxoEE-3

	CTT-HA	*B. subtilis*-CTT	*E. coli*-CTT	*B. subtilis*-TPM
***B. subtilis*-CTT**	prey presence (*Bs*)	-	-	-
***E. coli*-CTT**	prey presence (*Ec*)	prey identity	-	-
***B. subtilis*-TPM**	prey presence<br>casitone presence	casitone presence<br>prey-growth conditions[a]	prey identity<br>casitone presence<br>prey-growth conditions	-
***E. coli*-TPM**	prey presence<br>casitone presence	prey identity<br>casitone presence<br>prey-growth conditions	casitone presence<br>prey-growth conditions	prey identity

[a] *prey-growth conditions* refer to conditions for the period of overnight prey growth to stationary phase immediately prior to inoculation of MyxoEE-3 agar plates with *M. xanthus*. Prey growth during this period occurred either on the MyxoEE-3 agar plates themselves in the case of the *B. subtilis*-CTT and *E. coli*-CTT treatments or in CTT liquid (with an equivalent amount of casitone available as in the prey-CTT plates) prior to spreading on TPM starvation agar in the *B. subtilis*-TPM and *E. coli*-TPM treatments (see *Methods: MyxoEE-3*).

Supp. Table 3: Loci with mutations in clones from two or more of the TS-abiotic populations examined here. Populations (P) shown in red exhibited a significant decrease in fruiting body counts relative to the ancestor.

Old locus ID	New locus ID	annotated function	CTT-HA	CTT-SA
MXAN_6012	*MXAN_RS29170*	response regulator	P1, P3, P5, P7	P31, P33, P35, P37
frzF (MXAN_4138)	*MXAN_RS20090*	protein methyltransferase FrzF	P1, P3, P5, P9, P11	P29, P31, P33, P35, P37, P39
MXAN_5852	*MXAN_RS28370*	sensory box histidine kinase	-	P31, P33, P35, P37
lonD (MXAN_2017)	*MXAN_RS09780*	ATP-dependent protease LonD	-	P29, P33, P35, P37
MXAN_7216	*MXAN_RS34935*	ICE-like protease (caspase) p20 domain protein	P3, P7, P9, P11	-
hsfB (MXAN_5365)	*MXAN_RS26035*	response regulator/sensor histidine kinase HsfB	P1, P5, P9	-
MXAN_5032	*MXAN_RS24445*	efflux transporter, HAE1 family, inner membrane component	P5	P35, P37
MXAN_5030	*MXAN_RS24435*	efflux transporter, HAE1 family, outer membrane efflux protein	-	P31, P33
MXAN_0289	*MXAN_RS01415*	putative membrane protein	P3, P5	-

Supp. Table 4: Complete list of all the mutations previously identified in the TS-abiotic populations (ref. 79)

Treatment	Population	Position	Locus	Nuc. change	Mutation type	Mutation type	aa change
CTT-HA	P1	413,728	*rimK*	G-A	non-synon	transition	R222W
CTT-HA	P1	2,328,274	*rpsI*	A-T	non-synon	transversion	E17V
CTT-HA	P1	2,748,213	*glnS*	A-C	non-synon	transversion	Y419S
CTT-HA	P1	3,620,251	*MXAN_3092*	C-T	non-synon	transition	P1142L
CTT-HA	P1	3,931,943	*MXAN_3382*	C-T	synon	transition	G26G
CTT-HA	P1	4,560,157	*rpCTT-SA*	C-T	non-synon	transition	D479N
CTT-HA	P1	5,084,723	*frzF*	A-C	non-synon	transversion	I109S
CTT-HA	P1	6,679,133	*hsfB*	T-C	non-synon	transition	Y491C
CTT-HA	P1	6,831,379	*MXAN_5488*	C-T	synon	transition	Q302Q
CTT-HA	P1	7,442,735	*MXAN_6012*	2bp-AT	non-synon	Other	coding (318-319/375nt)
CTT-HA	P1	8,806,578	*MXAN_7214*	C-T	non-synon	transition	A63V
CTT-HA	P1	8,966,991	*rrsD/MXAN_7346*	T-C	intergenic	transition	intergenic (_273/+628)
CTT-HA	P3	345,175	*MXAN_0289*	C-T	stop	transition	Q691*
CTT-HA	P3	2,966,807	*pkn5*	C-T	non-synon	transition	E163K
CTT-HA	P3	3,590,850	*MXAN_3061*	T-C	non-synon	transition	V88A
CTT-HA	P3	4,493,896	*MXAN_3774*	G-T	non-synon	transversion	E462D
CTT-HA	P3	5,041,304	*dmpA*	A-C	non-synon	transversion	V63G
CTT-HA	P3	6,143,742	*MXAN_4910*	A-G	non-synon	transition	D2G
CTT-HA	P3	8,208,062	*MXAN_6679*	T-C	non-synon	transition	Y2665C
CTT-HA	P3	8,808,507	*MXAN_7216*	T-A	non-synon	transversion	W277R
CTT-HA	P5	659,229	*MXAN_0571*	G-A	synon	transition	L645L
CTT-HA	P5	2,803,548	*fuCTT-SA*	A-C	non-synon	transversion	F232V
CTT-HA	P5	3,107,730	*MXAN_2661*	G-T	non-synon	transversion	L136I
CTT-HA	P5	4,422,456	*MXAN_3708/ MXAN_3709*	G-T	intergenic	transversion	intergenic (_24/+87)
CTT-HA	P5	5,982,395	*MXAN_4784*	G-A	non-synon	transition	A344V
CTT-HA	P5	6,290,306	*MXAN_5032*	G-C	non-synon	transversion	A34P
CTT-HA	P5	7,070,726	*MXAN_5703*	G-A	synon	transition	L84L

CTT-HA	P5	7,166,230	*pilB*	T-A	non-synon	transversion	D206V
CTT-HA	P5	7,261,988	*MXAN_5852*	Δ1bp	frameshift	Deletion	coding (2891/2973nt)
CTT-HA	P5	8,809,349	*MXAN_7217*	A-C	non-synon	transversion	D69A
CTT-HA	P7	4,158,711	*MXAN_3570*	C-T	synon	transition	H35H
CTT-HA	P7	4,482,253	*MXAN_3764/era*	T-G	intergenic	transversion	intergenic (+108/_14)
CTT-HA	P7	5,088,813	*frzCD*	A-C	non-synon	transversion	S298A
CTT-HA	P7	9,062,185	*nla24*	C-T	non-synon	transition	R63C
CTT-HA	P9	865,861	*MXAN_0757*	G-A	synon	transition	L514L
CTT-HA	P9	4,456,661	*MXAN_3741*	G-A	noncoding	transition	noncoding (45/73nt)
CTT-HA	P9	5,950,201	*MXAN_4747*	A-C	non-synon	transversion	Y333S
CTT-HA	P9	6,289,148	*MXAN_5031*	T-G	non-synon	transversion	L33R
CTT-HA	P9	6,597,408	*MXAN_5295*	C-T	stop	transition	W293*
CTT-HA	P11	1,273,632	*MXAN_1093*	A-G	non-synon	transition	S53P
CTT-HA	P11	2,132,660	*MXAN_1807/MXAN_1808*	C-T	intergenic	transition	intergenic (_480/_23)
CTT-HA	P11	2,533,977	*MXAN_2194*	C-T	synon	transition	K125K
CTT-HA	P11	3,405,371	*pkn7*	T-G	non-synon	transversion	L119R
CTT-HA	P11	4,084,368	*MXAN_3505*	C-T	non-synon	transition	S12L
CTT-HA	P11	4,921,465	*MXAN_4017*	G-A	non-synon	transition	R145H
CTT-HA	P11	6,583,434	*MXAN_5280*	G-A	non-synon	transition	A55T
CTT-HA	P11	7,298,607	*MXAN_5879*	C-A	non-synon	transversion	P102H
CTT-HA	P11	8,045,866	*MXAN_6522/rpmG*	A-G	intergenic	transition	intergenic (+48/_115)
CTT-HA	P11	8,715,782	*MXAN_7139*	T-A	non-synon	transversion	L292Q
CTT-SA	P31	763,737	*MXAN_0664 / MXAN_0676*	Δ104041 bp	frameshift	Deletion	
CTT-SA	P31	1,620,347	*MXAN_1380*	C-T	stop	transition	Q22*
CTT-SA	P31	3,863,052	*rpsE*	G-A	non-synon	transition	E59K
CTT-SA	P31	4,696,495	*MXAN_3903*	G-A	stop	transition	Q30*
CTT-SA	P31	5,446,699	*MXAN_4411*	C-T	synon	transition	E10E
CTT-SA	P31	5,857,235	*MXAN_4674*	G-A	stop	transition	Q119*
CTT-SA	P31	5,887,434	*MXAN_4699*	A-G	non-synon	transition	I438T
CTT-SA	P31	5,998,849	*MXAN_4798*	C-T	synon	transition	P1039P

CTT-SA	P31	6,288,087	*MXAN_5030*	G-A	stop	transition	W147*
CTT-SA	P31	6,489,464	*MXAN_5196*	G-A	stop	transition	Q200*
CTT-SA	P31	6,653,911	*secA*	T-G	non-synon	transversion	D828A
CTT-SA	P31	8,100,723	*MXAN_6574/MXAN_6575*	G-A	intergenic	transition	intergenic (_147/+25)
CTT-SA	P33	770,850	*MXAN_0670*	A-G	non-synon	transition	S120P
CTT-SA	P33	3,757,099	*MXAN_3206/MXAN_3207*	C-A	intergenic	transversion	intergenic (_59/_453)
CTT-SA	P33	3,865,248	*secY*	C-T	synon	transition	T298T
CTT-SA	P33	4,091,120	*pyrC*	C-T	non-synon	transition	T91I
CTT-SA	P33	4,862,310	*lon*	C-T	stop	transition	Q70*
CTT-SA	P33	6,426,490	*cheB3*	A-G	non-synon	transition	L14P
CTT-SA	P33	7,771,923	*MXAN_6295*	G-A	noncoding	transition	noncoding (17/77nt)
CTT-SA	P35	150,453	*MXAN_0128*	Δ1bp	frameshift	Deletion	coding (1623/2040nt)
CTT-SA	P35	661,325	*MXAN_0575*	Δ1bp	frameshift	Deletion	coding (309/408nt)
CTT-SA	P35	1,319,525	*MXAN_1131*	Δ7bp	frameshift	Deletion	coding (525_531/1713nt)
CTT-SA	P35	2,220,932	*MXAN_1896*	A-C	non-synon	transversion	E662A
CTT-SA	P35	3,597,967	*rplJ*	A-C	non-synon	transversion	E27D
CTT-SA	P35	3,604,626	*rpoC*	A-T	non-synon	transversion	E403V
CTT-SA	P35	3,756,770	*MXAN_3206*	T-G	non-synon	transversion	t91P
CTT-SA	P35	4,135,872	*MXAN_3552*	C-T	non-synon	transition	P112L
CTT-SA	P35	4,178,849	*cspD*	T-G	non-synon	transversion	F17C
CTT-SA	P35	6,103,418	*MXAN_4870*	G-A	non-synon	transition	T41M
CTT-SA	P35	6,372,046	*MXAN_5098*	G-A	synon	transition	L116L
CTT-SA	P35	6,611,651	*MXAN_5307*	A-G	non-synon	transition	N78D
CTT-SA	P35	8,535,470	*MXAN_6975_/_MXAN_6976*	G-A	intergenic	transition	Intergenic (_200/_108)
CTT-SA	P35	8,913,768	*MXAN_7297_/_MXAN_7298*	T-C	intergenic	transition	Intergenic (+194/_187)
CTT-SA	P35	8,942,092	*MXAN_7321*	T-C	non-synon	transition	L120P
CTT-SA	P35	8,956,425	*MXAN_7332*	C-T	synon	transition	D215D
CTT-SA	P37	429,911	*MXAN_0362*	G-A	non-synon	transition	T162M
CTT-SA	P37	489,404	*MXAN_0429/MXAN_0430*	G-A	intergenic	transition	Intergenic (_946/+445)

CTT-SA	P37	1,276,210	*pyrG*	A-T	non-synon	transversion	F239M
CTT-SA	P37	1,446,423	*MXAN_1233*	C-G	non-synon	transversion	P407R
CTT-SA	P37	1,607,700	*MXAN_1366*	G-A	stop	transition	Q123*
CTT-SA	P37	4,023,450	*MXAN_3453*	C-T	non-synon	transition	R419H
CTT-SA	P37	4,811,949	*MXAN_3952*	G-C	non-synon	transversion	A390P
CTT-SA	P37	5,103,938	*MXAN_4155*	Δ 12bp	frameshift	Deletion	coding (1110_1121/2424nt)
CTT-SA	P37	7,779,370	*MXAN_6302*	T-G	non-synon	transversion	A29A
CTT-SA	P37	8,263,779	*MXAN_6720*	G-T	non-synon	transversion	K47N
CTT-SA	P39	770,098	*MXAN_0668*	Δ 1bp	frameshift	Deletion	Coding (14/813nt)
CTT-SA	P39	3,465,238	*MXAN_2956/MXAN_2958*	C-G	intergenic	transversion	Intergenic (+274/_775)
CTT-SA	P39	4,698,803	*MXAN_3905*	G-A	stop	transition	Q127*
CTT-SA	P39	4,939,747	*MXAN_4031*	T-C	non-synon	transition	L92P
CTT-SA	P39	7,158,747	*MXAN_5782*	G-C	non-synon	transversion	A11G
CTT-SA	P39	7,158,862	*pilA*	T-C	non-synon	transition	N200S
CTT-SA	P39	7,265,854	*acCTT-SA*	G-A	stop	transition	W51*
CTT-SA	P39	7,463,317	*MXAN_6031*	G-A	stop	transition	Q344*
CTT-SA	P39	7,490,691	*htpG*	C-G	synon	transversion	S497S
CTT-SA	P39	8,246,003	*MXAN_6704*	+G	frameshift	Insertion	coding (401/537nt)
